# Advanced MRI for targeting the pallidothalamic region in magnetic resonance-guided focused ultrasound: the mammillothalamic tract as an adjacent imaging landmark

**DOI:** 10.3389/fradi.2026.1856951

**Published:** 2026-06-24

**Authors:** Francisco Rivera, Nelson Quintanal Cordero, Fabián Piedimonte

**Affiliations:** Fundación CENIT para la Investigación en Neurociencias, Buenos Aires, Argentina

**Keywords:** diffusion tensor imaging, functional neurosurgery, magnetic resonance-guided focused ultrasound, mammillothalamic tract, pallidothalamic tractotomy, Parkinson's disease, stereotactic targeting

## Abstract

Magnetic resonance–guided focused ultrasound (MRgFUS) has expanded the therapeutic landscape for movement disorders, with growing interest in circuit-based targets such as pallidothalamic tractotomy (PTT) for Parkinson's disease. Structural MRI provides indirect orientation through surrounding landmarks, including the subthalamic nucleus, red nucleus, and internal capsule, while advanced sequences such as FGATIR and susceptibility-based imaging improve delineation of deep gray matter structures but remain limited for direct fiber visualization. Diffusion-based imaging offers complementary information by enabling *in vivo* reconstruction of white matter pathways, although tractography of pallidothalamic fibers remains constrained by crossing fiber architecture and spatial resolution. Within this context, the mammillothalamic tract represents a consistent and reproducible adjacent structure with reliable visibility on both structural and diffusion imaging. Its stable anatomical relationship to the pallidothalamic region supports its use as a practical landmark to refine stereotactic orientation when direct target visualization is not feasible. Together, these observations support a multimodal imaging framework that integrates structural and diffusion techniques to improve targeting accuracy in MRgFUS PTT. In this mini review, we aimed to synthesize the relevant anatomy of the pallidothalamic pathways and critically evaluate current MRI techniques for their localization. Additionally we sought to describe the role of identifying the mamillothalamic tract in MRgFUS-PTT planning.

## 1 Introduction

Magnetic resonance–guided focused ultrasound (MRgFUS) has emerged as a non-invasive ablative therapy for the treatment of movement disorders (1, 2). The technique enables stereotactic thermal lesioning under real-time MRI guidance without the need for intracranial instrumentation. Over the past decade, MRgFUS has become an established treatment for medication-refractory essential tremor (ET) and is increasingly being investigated for the management of Parkinson's disease (PD) (2–5). Several anatomical targets have been explored, including the ventral intermediate nucleus (VIM) of the thalamus, the subthalamic nucleus (STN), and pallidal pathways within the basal ganglia circuitry (1, 2, 4, 5).

Among emerging circuit-based targets, pallidothalamic tractotomy (PTT) has gained renewed interest as a strategy to interrupt pallidal efferent output before it reaches the motor thalamus. MRgFUS experiences suggest that lesioning this fiber convergence may improve tremor, rigidity, and hypokinetic motor features in selected patients with Parkinson's disease, although the available evidence remains limited and derives largely from single-center series (6, 7). Pallidal efferent fibers from the GPi course through the ansa lenticularis and the lenticular fasciculus, the latter traversing Forel's field H2. These bundles converge in the prerubral subthalamic region and continue as the fasciculus thalamicus within Forel's field H1 before entering the motor thalamus (7). Lesioning this compact fiber convergence aims to modulate basal ganglia–thalamocortical circuits implicated in the motor manifestations of PD. Early clinical experiences with MRgFUS PTT have reported improvement in tremor, rigidity, and other motor symptoms in patients with advanced disease (8, 9).

Accurate targeting of the pallidothalamic region remains technically challenging. The therapeutic target lies within the subthalamic area, where several critical structures converge within a narrow anatomical corridor (10). These include the subthalamic nucleus, internal capsule, zona incerta, and the mammillothalamic tract (MTT), which lies in close proximity to the pallidothalamic fibers (7). Because the pallidothalamic tracts are small and difficult to visualize directly on conventional MRI, stereotactic targeting has historically relied on indirect anatomical landmarks derived from atlases and structural MRI. Advances in neuroimaging, particularly diffusion tensor imaging (DTI) and tractography, have enabled *in vivo* reconstruction of deep white matter pathways and have renewed interest in tract-informed targeting strategies (11). High-resolution diffusion imaging studies have demonstrated that the mammillothalamic tract can be reconstructed reliably *in vivo*, suggesting that it may serve as a useful imaging landmark in the subthalamic region (12). The mammillothalamic tract may offer a practical neighboring landmark for stereotactic orientation, but its role in MRgFUS PTT planning has not been synthesized clearly in the imaging literature.

Although the pallidothalamic region is an increasingly relevant target for MRgFUS, direct imaging of the target remains challenging. The potential role of the mammillothalamic tract as an adjacent imaging landmark for MRgFUS pallidothalamic tractotomy has not been synthesized clearly in the neuroimaging literature. In this mini review, we summarize the relevant anatomy of the pallidothalamic pathways and the mammillothalamic tract, and discuss MRI techniques that may assist in indirect localization of the pallidothalamic target region.

## 2 Anatomy of the pallidothalamic pathways

The pallidothalamic tract constitutes the principal efferent pathway of the internal segment of the globus pallidus (GPi), transmitting inhibitory basal ganglia output to the motor thalamus (13–16). Two major fiber bundles arise from the GPi: the ansa lenticularis and the lenticular fasciculus (13, 14, 16). The ansa lenticularis emerges from the ventromedial GPi and courses medially and inferiorly around the internal capsule before turning dorsally toward the thalamus (13). In contrast, the lenticular fasciculus originates from the dorsomedial GPi and penetrates the internal capsule more directly, traveling superior to the subthalamic nucleus (13). These pathways converge in the prerubral subthalamic region before ascending as the fasciculus thalamicus within Forel's field H1, which projects to the ventral anterior and ventrolateral nuclei of the thalamus (7).

The convergence of pallidal efferent fibers within Forel's field H1 creates a compact anatomical region that is particularly relevant for functional lesioning procedures (7, 13). Interruption of these fibers forms the basis of PTT, which aims to modulate basal ganglia output before thalamic entry (7, 17). Clinical studies using MRgFUS have reported improvement in tremor, rigidity, and bradykinesia following lesioning of this region in patients with advanced Parkinson's disease (8, 18). However, the pallidothalamic region lies within a complex subthalamic corridor that also contains the subthalamic nucleus, zona incerta, internal capsule, and the mammillothalamic tract. These structures are separated by only a few millimeters, making precise stereotactic localization essential (18–20). Given that the pallidothalamic fibers are not consistently visualized on conventional MRI sequences, targeting strategies often rely on indirect anatomical landmarks derived from stereotactic atlases and structural imaging (19, 21). Among these landmarks, the MTT has emerged as a particularly useful neighboring structure because of its relatively consistent anatomical position and its visibility on structural and diffusion MRI.

## 3 Mammillothalamic tract: anatomy and imaging visibility

The MTT is a compact white matter pathway that connects the mammillary bodies of the hypothalamus to the anterior nuclei of the thalamus and forms an important component of the Papez circuit involved in memory processing (14, 22). The MTT originates in the mammillary bodies, courses superiorly through the hypothalamic region, and ascends to the anterior thalamic nuclei as a key component of the Papez circuit (22, 23). In the subthalamic region, the MTT lies medial to the pallidothalamic fibers and anterior to the subthalamic nucleus, placing it in close proximity to the stereotactic targets used for pallidothalamic tractotomy (23). Because of this spatial relationship, the MTT represents an important neighboring structure that must be considered during procedures targeting the pallidothalamic region.

**Figure 1:**
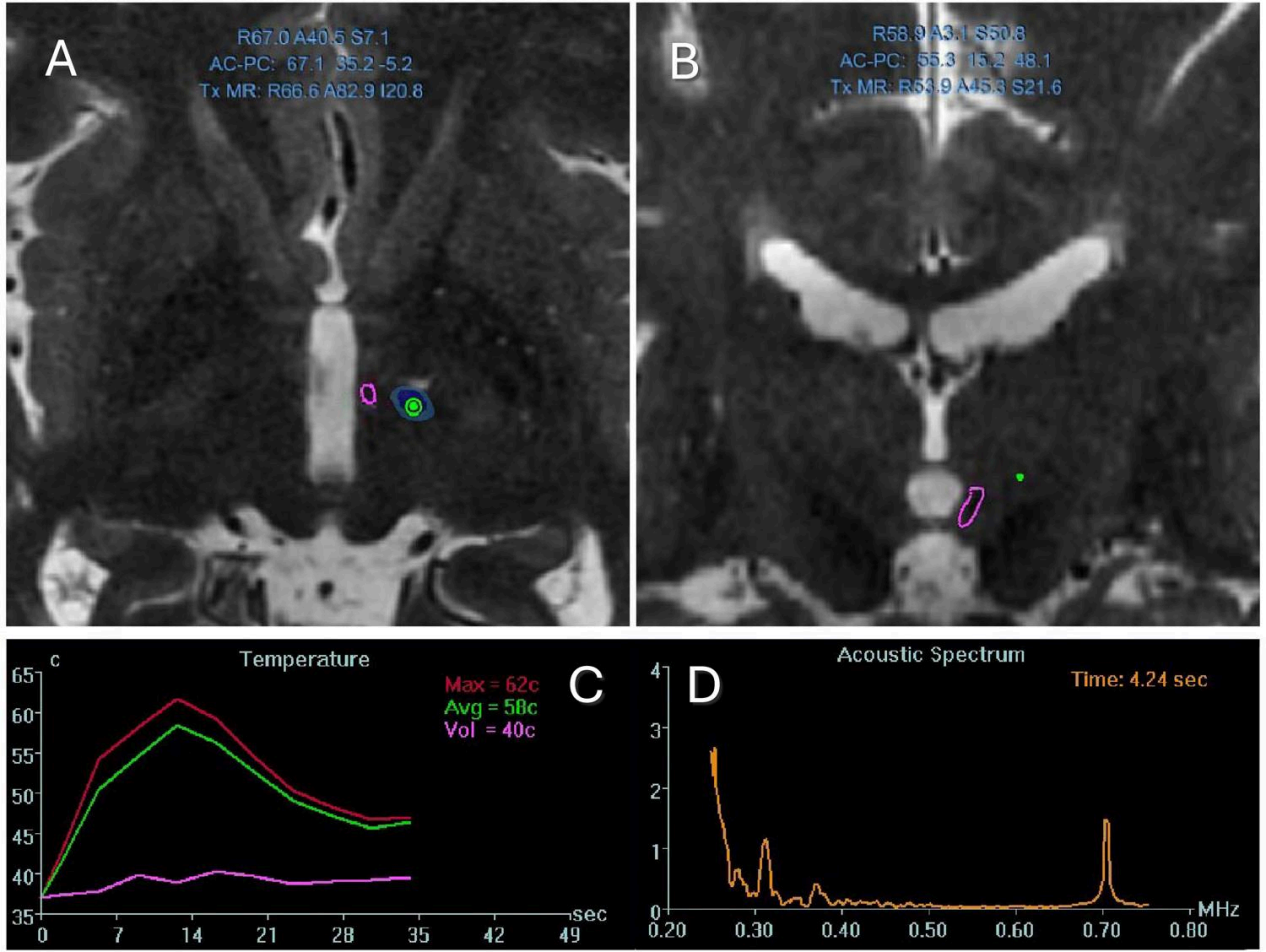
Clinical example of imaging-based mammillothalamic tract localization and thermal monitoring during MRgFUS pallidothalamic tractotomy (PTT). **(A)** Axial T2-weighted MRI demonstrating the mammillothalamic tract (MTT) highlighted in magenta. The planned PTT target is depicted as a green circular region, while the corresponding thermal lesion (thermal focal spot) is shown as a blue-background area. **(B)** Coronal MRI illustrating the MTT in magenta and the planned PTT target as a focal green point. **(C)** Temperature profile recorded during sonication, showing peak temperatures at the PTT target (green) and within the MTT region (magenta). **(D)** Acoustic spectrum recorded during treatment.

Compared with the pallidothalamic fibers, which remain difficult to visualize directly with conventional MRI sequences, the mammillothalamic tract has been shown to be identifiable using advanced imaging techniques. Early MRI studies demonstrated that the MTT can be visualized on high-resolution structural imaging due to its compact fiber organization and consistent anatomical course (24). More recently, DTI and tractography have enabled *in vivo* reconstruction of the tract and characterization of its trajectory from the mammillary bodies to the anterior thalamus (12). The relatively consistent anatomy and imaging visibility of the MTT therefore make it a useful stereotactic landmark in the subthalamic region, particularly when direct visualization of adjacent fiber tracts such as the pallidothalamic tracts is limited. In clinical planning, the MTT can be identified as a compact tract extending from the mammillary bodies toward the anterior thalamus, using high-resolution structural MRI and, when available, diffusion-based tractography to confirm its trajectory. An example of MTT localization during MRgFUS-PTT can be found in Figure 1.

## 4 Advanced MRI for indirect localization of the pallidothalamic target region

Accurate localization of the pallidothalamic region remains challenging because the pallidothalamic fibers are small and are not consistently visualized on conventional structural MRI (7, 25). As a result, stereotactic targeting has traditionally relied on indirect anatomical landmarks derived from atlas-based coordinates and standard T1- and T2-weighted imaging (19, 26). These sequences allow identification of major deep brain structures such as the subthalamic nucleus, red nucleus, and internal capsule, which serve as reference points for targeting the subthalamic region (27). Traditionally, PTT target setting has relied on atlas-based stereotactic planning using the anterior commissure–posterior commissure line, the midcommissural point, and standard structural MRI landmarks, including the thalamus, red nucleus, subthalamic nucleus, and internal capsule. In this conventional approach, MRI is primarily used to confirm patient-specific anatomy and avoid adjacent critical structures, while the pallidothalamic fibers themselves remain indirectly inferred rather than directly visualized. In MRgFUS PTT, Gallay et al. described targeting based on the intercommissural line and thalamo-ventricular border, with the target center located approximately 6.5 mm lateral to the medial thalamic border, 1 mm posterior to the midcommissural line, and on the intercommissural plane (6). These coordinates provide a practical stereotactic framework but require adjustment according to patient-specific anatomy. Incorporating the MTT does not replace these conventional coordinates; rather, it adds a patient-specific anatomical reference that can be used to verify the medial and anterior boundaries of the target region. Practically, the planned PTT target can first be selected using conventional AC-PC-based coordinates and then refined by confirming its spatial relationship to the visible MTT on structural MRI or diffusion-based tractography. However, the pallidothalamic tracts themselves are rarely distinguishable on routine MRI because of their small caliber and their location within a densely packed fiber region of the subthalamic area. In this context, the identification of neighboring anatomical landmarks becomes particularly important for stereotactic orientation. Rather than representing the therapeutic target itself, the mammillothalamic tract may serve as a visible adjacent structure that helps define the spatial organization of the subthalamic region when direct visualization of pallidothalamic fibers is limited. Because the mammillothalamic tract follows a relatively consistent anatomical trajectory and can be reconstructed using diffusion-based imaging techniques, it may assist in improving anatomical orientation during MRgFUS pallidothalamic tractotomy planning.

Recent advances in MRI acquisition have improved the visualization of deep brain structures relevant to functional neurosurgery. High-resolution sequences, including proton density imaging and FGATIR, enhance gray–white matter contrast in the subthalamic region and improve identification of anatomical landmarks (27, 28). Susceptibility-based imaging, including SWI and QSM, enhances visualization of iron-rich structures such as the subthalamic nucleus and substantia nigra for stereotactic planning (29). These techniques have primarily been applied to the delineation of deep gray matter nuclei rather than to the visualization of small white matter pathways such as the pallidothalamic or mammillothalamic tracts. Diffusion-based imaging methods have also emerged as an important tool for visualizing white matter pathways relevant to functional neurosurgery. Diffusion tensor imaging and tractography enable *in vivo* reconstruction of major white matter pathways and are increasingly used to guide targeting in deep brain stimulation and lesioning procedures (12, 30). Direct tractography of the pallidothalamic region remains challenging because of crossing fibers and limited spatial resolution (31, 32). However, diffusion imaging can reliably reconstruct neighboring tracts such as the mammillothalamic pathway (12). Combined with high-resolution structural imaging, tractography may therefore support a multimodal approach for identifying the pallidothalamic region and improving targeting accuracy in MRgFUS pallidothalamic tractotomy. The different techniques used to localize the pallidothalamic region and their characteristics are shown in Table 1.

**Table 1:** MRI techniques relevant for indirect localization of the pallidothalamic region and visualization of adjacent anatomical landmarks.

MRI technique	Structures visualized	Advantages	Limitations
T1-weighted MRI	General deep brain anatomy (thalamus, basal ganglia)	Widely available; used for stereotactic planning	Limited contrast for small fiber pathways
T2-weighted MRI	Subthalamic nucleus, red nucleus, surrounding landmarks	Good anatomical orientation for deep brain structures	Direct visualization of small fiber tracts remains limited
Proton density imaging	Subthalamic region structures	Improved contrast in deep brain nuclei	Limited tract visualization
FGATIR (Fast Gray Matter Acquisition T1 Inversion Recovery)	Subthalamic nucleus and adjacent gray matter structures	Enhanced gray–white matter contrast in deep brain regions	Limited evidence for direct pallidothalamic tract visualization
SWI/QSM	Iron-rich structures (substantia nigra, STN)	Improved delineation of subcortical nuclei	Fiber tracts not reliably visualized
Diffusion Tensor Imaging (DTI)	White matter pathways including mammillothalamic tract	Enables tractography and fiber reconstruction	Sensitive to crossing fibers and spatial resolution limits

T1-weighted MRI, T1-weighted magnetic resonance imaging; T2-weighted MRI, T2-weighted magnetic resonance imaging; FGATIR, Fast Gray Matter Acquisition T1 Inversion Recovery; SWI, susceptibility-weighted imaging; QSM, quantitative susceptibility mapping; STN, subthalamic nucleus; DTI, diffusion tensor imaging.

## 5 Discussion

This mini review highlights the potential role of advanced MRI in improving anatomical orientation of the pallidothalamic region for MRgFUS pallidothalamic tractotomy. We emphasize the mammillothalamic tract as a practical adjacent imaging landmark. MRgFUS has expanded the range of non-invasive therapeutic options for movement disorders. While thalamotomy targeting the ventral intermediate nucleus remains the most established application, increasing attention has been directed toward alternative circuit-based targets within the basal ganglia network. Pallidothalamic tractotomy represents one such strategy, aiming to interrupt the pathological output of the internal segment of the globus pallidus before its projection to the motor thalamus. Early clinical experiences with MRgFUS PTT have demonstrated meaningful improvements in tremor and other motor symptoms in patients with advanced Parkinson's disease (6, 8). However, accurate targeting of this region remains technically demanding because the pallidothalamic fibers lie within a densely packed subthalamic fiber tracts that cannot be consistently visualized using conventional structural MRI.

The anatomical complexity of the subthalamic region underscores the importance of reliable imaging landmarks during stereotactic procedures. Within this context, the mammillothalamic tract represents a particularly relevant structure. Its compact organization and relatively consistent trajectory make it more readily identifiable on structural and diffusion imaging compared with neighboring pallidothalamic fibers. Diffusion-based studies have demonstrated reproducible *in vivo* reconstruction of the MTT, supporting its potential value as an anatomical reference for procedures targeting adjacent fiber tracts (12). Identification of the MTT may therefore assist in defining the medial boundary of the pallidothalamic region and improving anatomical orientation during MRgFUS planning. However, inter-subject variability in subthalamic anatomy may alter the spatial relationship between the MTT and the pallidothalamic target, supporting the need for patient-specific MRI-based planning rather than reliance on atlas coordinates alone (25, 33). From a practical perspective, the value of the MTT in this setting lies not in representing the therapeutic target itself but in providing a reproducible neighboring landmark within a complex anatomical corridor. In procedures such as MRgFUS pallidothalamic tractotomy, where millimetric targeting accuracy is required and direct visualization of the target fibers remains limited, identification of adjacent structures may contribute to more reliable spatial orientation during stereotactic planning. By providing a visible adjacent landmark, MTT identification may improve confidence that the planned lesion remains within the intended subthalamic corridor and may help avoid unintended medial extension toward memory-related circuitry. However, whether MTT-referenced planning improves targeting accuracy or reduces adverse events compared with conventional atlas-based targeting remains to be demonstrated in future clinical studies. Notably, the MTT is a key component of the Papez circuit, inadvertent thermal injury to this pathway may theoretically produce memory-related adverse effects, including anterograde memory impairment or amnestic symptoms (34). Therefore, in MRgFUS-PTT planning, the MTT should be considered a structure to identify and preserve.

Current targeting strategies for pallidothalamic tractotomy continue to rely on a combination of stereotactic atlas coordinates and indirect MRI landmarks. High-resolution structural sequences allow visualization of surrounding nuclei such as the subthalamic nucleus, red nucleus, and internal capsule, which serve as key spatial references during treatment planning. Advanced MRI techniques may further improve this process. Inversion-recovery sequences such as FGATIR enhance contrast within deep brain structures, while susceptibility-based imaging improves delineation of iron-rich nuclei. Diffusion-based tractography offers additional information by reconstructing neighboring white matter pathways, including the mammillothalamic tract. Although direct tractography of pallidothalamic fibers remains limited by crossing fiber architecture and spatial resolution constraints, the integration of structural and diffusion imaging may provide a multimodal framework for identifying the pallidothalamic region during MRgFUS procedures.

Despite these advances, several challenges remain. Variability in MRI acquisition protocols, tractography algorithms, and stereotactic planning methods currently limits standardization across institutions. In addition, diffusion-based reconstruction of small fiber tracts within the subthalamic region remains technically challenging. Future developments in high-resolution diffusion imaging, multi-shell diffusion models, and ultra-high-field MRI may improve the visualization of these pathways. Integration of structural imaging, tractography, and stereotactic atlases may ultimately enable more individualized targeting strategies for MRgFUS pallidothalamic tractotomy. Further studies correlating imaging findings with procedural accuracy and clinical outcomes will be essential to determine the optimal neuroimaging approaches for targeting this complex region.

## 6 Limitations

This mini review has limitations. The available literature specifically addressing MRgFUS pallidothalamic tractotomy remains limited, and much of the anatomical and imaging rationale is extrapolated from cadaveric studies, tractography work, and broader functional neurosurgical imaging literature. Therefore, at present, the MTT should not be used as a fixed millimetric offset for target selection, but as an adjunctive landmark to contextualize the planned lesion relative to surrounding patient-specific anatomy. Technical MRI parameters, including diffusion directions, b-values, voxel size, FGATIR acquisition parameters, and SWI/QSM echo and reconstruction methods, vary across studies and are not consistently reported, limiting direct comparison between imaging techniques. In addition, the available literature does not yet define validated quantitative distance thresholds between the MTT and the pallidothalamic target, nor does it establish correlations between MTT-based targeting and clinical outcomes. Direct MRI visualization of pallidothalamic fibers remains technically challenging, and currently available imaging approaches are best interpreted as complementary tools for indirect anatomical localization rather than definitive target delineation.

## 7 Conclusion

MRgFUS pallidothalamic tractotomy is an emerging circuit-based therapeutic strategy for Parkinson's disease, but accurate targeting of the pallidothalamic region remains challenging because the target fibers are not consistently visualized on conventional MRI and lie within a densely organized subthalamic corridor. Advanced neuroimaging, particularly high-resolution structural MRI and diffusion-based techniques, may improve indirect anatomical localization of this region. Within this framework, the mammillothalamic tract appears to be a practical adjacent landmark that may support stereotactic orientation during MRgFUS pallidothalamic tractotomy planning. Further studies correlating imaging findings with targeting accuracy and clinical outcomes will be necessary to define the optimal imaging approach for this region.

## References

[1] Martínez-Fernández R Rodríguez-Rojas R Del Álamo M Hernández-Fernández F Pineda-Pardo JA Dileone M. Focused ultrasound subthalamotomy in patients with asymmetric Parkinson's disease: a pilot study. Lancet Neurol. (2018) 17(1):54–63. 10.1016/S1474-4422(17)30403-9 29203153

[2] Martínez-Fernández R Máñez-Miró JU Rodríguez-Rojas R Del Álamo M Shah BB Hernández-Fernández F. Randomized trial of focused ultrasound subthalamotomy for Parkinson's disease. N Engl J Med. (2020) 383(26):2501–13. 10.1056/NEJMoa2016311 33369354

[3] Arcadi A Aviles-Olmos I Gonzalez-Quarante LH Gorospe A Jiménez-Huete A de la Corte MM. Magnetic resonance-guided focused ultrasound (MRgFUS)-thalamotomy for essential tremor: lesion location and clinical outcomes. Mov Disord. (2024) 39(6):1015–25. 10.1002/mds.29801 38616324

[4] Rivera F Cordero NEQ Miranda RB Piedimonte F. Palidotomía mediante ultrasonido focalizado guiado por resonancia magnética en la enfermedad de Parkinson: revisión sistemática de eficacia y seguridad. NeuroTarget. (2024) 18(1):78–84. 10.47924/neurotarget2024472

[5] Miranda RB Rivera F Cordero NEQ Piedimonte F. Talamotomía VIM con RMgFUS en la enfermedad de Parkinson: eficacia, seguridad y técnica. NeuroTarget. (2025) 19(1):48–54. 10.47924/neurotarget2025481

[6] Gallay MN Moser D Rossi F Magara AE Strasser M Bühler R. MRgFUS pallidothalamic tractotomy for chronic therapy-resistant Parkinson's disease in 51 consecutive patients: single center experience. Front Surg. (2019) 6:76. 10.3389/fsurg.2019.00076 31993437 PMC6971056

[7] Gallay MN Jeanmonod D Liu J Morel A. Human pallidothalamic and cerebellothalamic tracts: anatomical basis for functional stereotactic neurosurgery. Brain Struct Funct. (2008) 212(6):443–63. 10.1007/s00429-007-0170-0 18193279 PMC2494572

[8] Gallay MN Moser D Jeanmonod D. Safety and accuracy of incisionless transcranial MR-guided focused ultrasound functional neurosurgery: single-center experience with 253 targets in 180 treatments. J Neurosurg. (2019) 130(4):1234–43. 10.3171/2017.12.JNS172054 29799340

[9] Gallay MN Moser D Magara AE Haufler F Jeanmonod D. Bilateral MR-guided focused ultrasound pallidothalamic tractotomy for Parkinson's disease with 1-year follow-up. Front Neurol. (2021) 12:601153. 10.3389/fneur.2021.601153 33633664 PMC7900542

[10] Horisawa S Kohara K Nonaka T Fukui A Mochizuki T Iijima M. Unilateral pallidothalamic tractotomy at Forel's field H1 for cervical dystonia. Ann Clin Transl Neurol. (2022) 9(4):478–87. 10.1002/acn3.51532 35261204 PMC8994978

[11] Jakab A Werner B Piccirelli M Kovács K Martin E Thornton JS. Feasibility of diffusion tractography for the reconstruction of intra-thalamic and cerebello-thalamic targets for functional neurosurgery: a multi-vendor pilot study in four subjects. Front Neuroanat. (2016) 10:76. 10.3389/fnana.2016.00076 27462207 PMC4940380

[12] Kamali A Zhang CC Riascos RF Tandon N Bonafante-Mejia EE Patel R. Diffusion tensor tractography of the mammillothalamic tract in the human brain using a high spatial resolution DTI technique. Sci Rep. (2018) 8(1):5229. 10.1038/s41598-018-23452-w 29588461 PMC5869722

[13] Parent A Hazrati LN. Functional anatomy of the basal ganglia. I. The cortico-basal ganglia-thalamo-cortical loop. Brain Res Rev. (1995) 20(1):91–127. 10.1016/0165-0173(94)00007-C 7711769

[14] Nieuwenhuys R Voogd J Huijzen C. The Human Central Nervous System. 4th ed. Vol. 29. Heidelberg: Springer (2008).

[15] Alexander GE DeLong MR Strick PL. Parallel organization of functionally segregated circuits linking basal ganglia and cortex. Annu Rev Neurosci. (1986) 9:357–81. 10.1146/annurev.ne.09.030186.002041 3085570

[16] Lanciego JL Luquin N Obeso JA. Functional neuroanatomy of the basal ganglia. Cold Spring Harb Perspect Med. (2012) 2(12):a009621. 10.1101/cshperspect.a009621 23071379 PMC3543080

[17] Coenen VA Mädler B Schiffbauer H Urbach H Allert N. Individual fiber anatomy of the subthalamic region revealed with diffusion tensor imaging: a concept to identify the deep brain stimulation target for tremor suppression. Neurosurgery. (2011) 68(4):1069–75. 10.1227/NEU.0b013e31820a1a20 21242831

[18] Chen JC Chen CM Aoh Y Cheng YK Lu MK Tsai CH. Immediate motor control enhancement via pallidothalamic tract (PTT) circuit ablation: a dual-target MR-guided focused ultrasound approach for tremor-dominant Parkinson's disease. Eur J Neurol. (2025) 32(9):e70345. 10.1111/ene.70345 40888435 PMC12400157

[19] Schaltenbrand G Wahren W. Atlas for Stereotaxy of the Human Brain: With an Accompanying Guide. Stuttgart: Thieme (1977).

[20] Chung BS Park JS. Whole course of pallidothalamic tracts identified on the sectioned images and surface models. Clin Anat. (2020) 33(1):66–76. 10.1002/ca.23468 31573101

[21] Horisawa S Miyao S Hori T Kohara K Kawamata T Taira T. Comorbid seizure reduction after pallidothalamic tractotomy for movement disorders: revival of Jinnai's Forel-H-tomy. Epilepsia Open. (2021) 6(1):225–9. 10.1002/epi4.12467 33681665 PMC7918322

[22] Catani M de Schotten MT. Atlas of Human Brain Connections. London, England: Oxford University Press (2012). 544 p.

[23] Giampiccolo D Duncan JS van Dijk J Baruteau KP Davagnanam I. Mammillothalamic tract. In: Duncan JS, Giampiccolo D, editors. MRI Neuroanatomy: Cortex, Nuclei and Connections. Cham: Springer Nature Switzerland AG (2025). p. 2665–71.

[24] Yamada K Shrier DA Rubio A Yoshiura T Iwanaga S Shibata DK. MR imaging of the mamillothalamic tract. Radiology. (1998) 207(3):593–8. 10.1148/radiology.207.3.9609878 9609878

[25] Plantinga BR Temel Y Duchin Y Uludağ K Patriat R Roebroeck A. Individualized parcellation of the subthalamic nucleus in patients with Parkinson's disease with 7T MRI. Neuroimage. (2018) 168:403–11. 10.1016/j.neuroimage.2016.09.023 27688203 PMC5479742

[26] Chen SY Tsai ST Hung HY Lin SH Pan YH Lin SZ. Targeting the subthalamic nucleus for deep brain stimulation–a comparative study between magnetic resonance images alone and fusion with computed tomographic images. World Neurosurg. (2011) 75(1):132–7. 10.1016/j.wneu.2010.09.009 21492677

[27] Sudhyadhom A Haq IU Foote KD Okun MS Bova FJ. A high resolution and high contrast MRI for differentiation of subcortical structures for DBS targeting: the fast gray matter acquisition T1 inversion recovery (FGATIR). Neuroimage. (2009) 47 (Suppl 2):T44–52. 10.1016/j.neuroimage.2009.04.018 19362595

[28] Tourdias T Saranathan M Levesque IR Su J Rutt BK. Visualization of intra-thalamic nuclei with optimized white-matter-nulled MPRAGE at 7T. Neuroimage. (2014) 84:534–45. 10.1016/j.neuroimage.2013.08.069 24018302 PMC3927795

[29] Deistung A Schäfer A Schweser F Biedermann U Turner R Reichenbach JR. Toward *in vivo* histology: a comparison of quantitative susceptibility mapping (QSM) with magnitude-, phase-, and R2*-imaging at ultra-high magnetic field strength. Neuroimage. (2013) 65:299–314. 10.1016/j.neuroimage.2012.09.055 23036448

[30] Sammartino F Krishna V King NKK Lozano AM Schwartz ML Huang Y. Tractography-based ventral intermediate nucleus targeting: novel methodology and intraoperative validation. Mov Disord. (2016) 31(8):1217–25. 10.1002/mds.26633 27214406 PMC5089633

[31] Jbabdi S Johansen-Berg H. Tractography: where do we go from here? Brain Connect. (2011) 1(3):169–83. 10.1089/brain.2011.0033 22433046 PMC3677805

[32] Maier-Hein KH Neher PF Houde JC Côté MA Garyfallidis E Zhong J. Author correction: the challenge of mapping the human connectome based on diffusion tractography. Nat Commun. (2019) 10(1):5059. 10.1038/s41467-019-12867-2 31685826 PMC6828749

[33] Ghaderi Niri S Khalaf AM Massoud TF. The mammillothalamic tracts: age-related conspicuity and normative morphometry on brain magnetic resonance imaging. Clin Anat. (2020) 33(6):911–9. 10.1002/ca.23595 32239548

[34] Aggleton JP Brown MW. Episodic memory, amnesia, and the hippocampal-anterior thalamic axis. Behav Brain Sci. (1999) 22(3):425–44. 10.1017/S0140525X99002034 11301518

